# Evidence of Field-Evolved Resistance to Bifenthrin in Western Corn Rootworm (*Diabrotica virgifera virgifera* LeConte) Populations in Western Nebraska and Kansas

**DOI:** 10.1371/journal.pone.0142299

**Published:** 2015-11-13

**Authors:** Adriano E. Pereira, Haichuan Wang, Sarah N. Zukoff, Lance J. Meinke, B. Wade French, Blair D. Siegfried

**Affiliations:** 1 Department of Entomology, University of Nebraska-Lincoln, Lincoln, Nebraska, United States of America; 2 Southwest Research and Extension Center, Kansas State University, Garden City, Kansas, United States of America; 3 North Central Agricultural Research Laboratory, United States Department of Agriculture-Agriculture Research Service, Brookings, South Dakota, United States of America; Instituto de Biotecnología, Universidad Nacional Autónoma de México, MEXICO

## Abstract

Pyrethroid insecticides have been used to control larvae or adults of the western corn rootworm (WCR), *Diabrotica virgifera virgifera* LeConte, a key pest of field corn in the United States. In response to reports of reduced efficacy of pyrethroids in WCR management programs in southwestern areas of Nebraska and Kansas the present research was designed to establish a baseline of susceptibility to the pyrethroid insecticide, bifenthrin, using susceptible laboratory populations and to compare this baseline with susceptibility of field populations. Concentration-response bioassays were performed to estimate the baseline susceptibility. From the baseline data, a diagnostic concentration (LC_99_) was determined and used to test adults of both laboratory and field populations. Larval susceptibility was also tested using both laboratory and field populations. Significant differences were recorded in adult and larval susceptibility among WCR field and laboratory populations. The highest LC_50_ for WCR adults was observed in populations from Keith 2 and Chase Counties, NE, with LC_50_s of 2.2 and 1.38 μg/vial, respectively, and Finney County 1, KS, with 1.43 μg/vial, as compared to a laboratory non-diapause population (0.24 μg/vial). For larvae, significant differences between WCR field and laboratory populations were also recorded. Significant differences in mortalities at the diagnostic bifenthrin concentration (LC_99_) were observed among WCR adult populations with western Corn Belt populations exhibiting lower susceptibility to bifenthrin, especially in southwestern Nebraska and southwestern Kansas. This study provides evidence that resistance to bifenthrin is evolving in field populations that have been exposed for multiple years to pyrethroid insecticides. Implications to sustainable rootworm management are discussed.

## Introduction

Considered the most important and challenging corn pest in the United States Corn Belt, the western corn rootworm (WCR), *Diabrotica virgifera virgifera* LeConte, has been estimated to cost corn growers over $1 billion in yield loss and control expenditures annually [1,2]. Development of behavioral resistance to crop rotation in the eastern Corn Belt [3] and resistance to some Cry toxins expressed in corn hybrids [4–6], as well as the introduction of this pest into Europe during the early 1990s [7] have increased management challenges associated with this pest. Damage to corn is caused by larvae feeding on the roots which compromises water and nutrient uptake [8,9] and may cause substantial reductions in grain yield [2,10]. At high infestation levels, damaged plants become lodged during strong rain or wind events making the plants difficult to harvest [11,12].

Several control methods have been used to suppress corn rootworm populations. Crop rotation with non-host plants has been an effective method because larvae are unable to develop on plants other than corn and a few native grass species [13–15]. However, behavioral resistance in WCR has been reported since the late 1980s and mid 1990s in the eastern Corn Belt [3,16,17], in which adult females exhibit reduced ovipositional fidelity to corn and oviposit a significant number of eggs in surrounding crops [3,18–20]. This behavior enables the WCR to circumvent crop rotation as a management tactic.

Insecticides have been used for corn rootworm larval control since the late 1940s when DDT and benzene hexachloride (BHC) were first introduced as soil treatments followed by aldrin and dieldrin [21–24]. Adulticides, such as organophosphates and carbamates, have been used in some areas to suppress WCR females and reduce egg laying [25]. The first case of resistance to insecticides in WCR was noted in 1959 in Nebraska to cyclodiene insecticides such as aldrin and heptachlor [23,24]. Cyclodiene resistance in WCR has persisted for more than 40 years throughout most of its distribution despite the U.S. ban of these insecticides in the early 1970s [26–28].

Organophosphates, carbamates, and pyrethroids replaced organochlorine insecticides as soil insecticides after cyclodiene resistance had become widespread [29]. In some regions of Nebraska, large areas adopted an adult management approach to control WCR ovipositing females [30,31] relying primarily on carbamate and organophosphate insecticides. Since then, the evolution of resistance in adult WCR to both insecticides has been reported in populations from different areas in Nebraska [30] and Kansas [32,33]. The use of both insecticide classes has since been restricted for corn rootworm management due to their common mode of action as acetylcholinesterase inhibitors and potential risks to human health [34–36].

The adoption of Bt crops has been beneficial in terms of reduced use of broad spectrum neurotoxic insecticides and reduced impact to non-target organisms [37,38]. Since 2003, transgenic corn hybrids expressing *Bacillus thuringiensis* endotoxins have been introduced [6,39] for rootworm control. However, resistance in WCR to corn hybrids expressing Cry3Bb1 toxins has been reported since 2009 in Iowa and other states [4,6,40,41] which confers cross-resistance to mCry3A expressing hybrids [5,6].

Pyrethroid insecticides remain one of the main chemical options to control corn rootworms both as soil insecticides which target rootworm larvae and as adulticides to prevent oviposition resulting in their widespread use, sometimes with multiple applications in a single growing season. In certain areas of western Nebraska and southwestern Kansas, reports of inadequate rootworm control with the pyrethroid insecticide, bifenthrin, have been increasing in recent years (SNZ and LJM, personal communication).

Because of the widespread use of bifenthrin as a soil insecticide and adulticide, the objectives of this research were to: 1) establish a baseline of susceptibility for WCR adults and larvae from lab populations and determine a diagnostic concentration (LC_99_) for adults, 2) determine susceptibility of field-collected populations throughout the U.S. Corn Belt by comparing survival of adults from field populations at a diagnostic concentration derived from baseline studies, and 3) compare susceptibility of WCR neonates between lab and field populations to the pyrethroid insecticide, bifenthrin.

## Materials and Methods

### WCR populations

**Fig 1:**
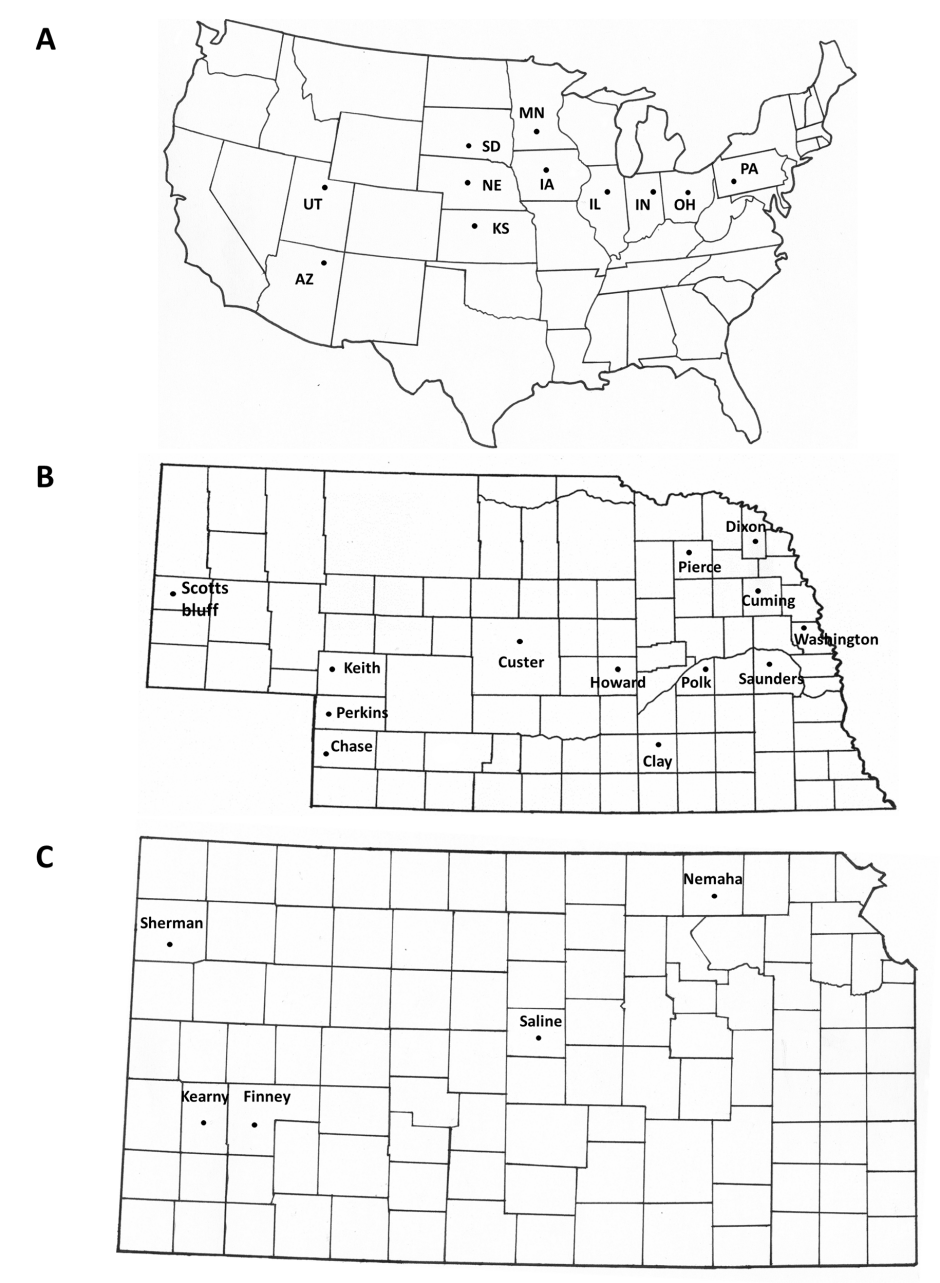
(A) U.S. map showing the 10 states where WCR field populations were collected, plus non-diapause laboratory colony (Brookings, South Dakota). **(B)** Nebraska state map showing 13 sites where field populations were collected. **(C)** Kansas state map showing five sites where field populations were collected. (Finney populations were collected in 2013 and 2014; Sherman and Saline populations were collected in 2014).

**Fig 2:**
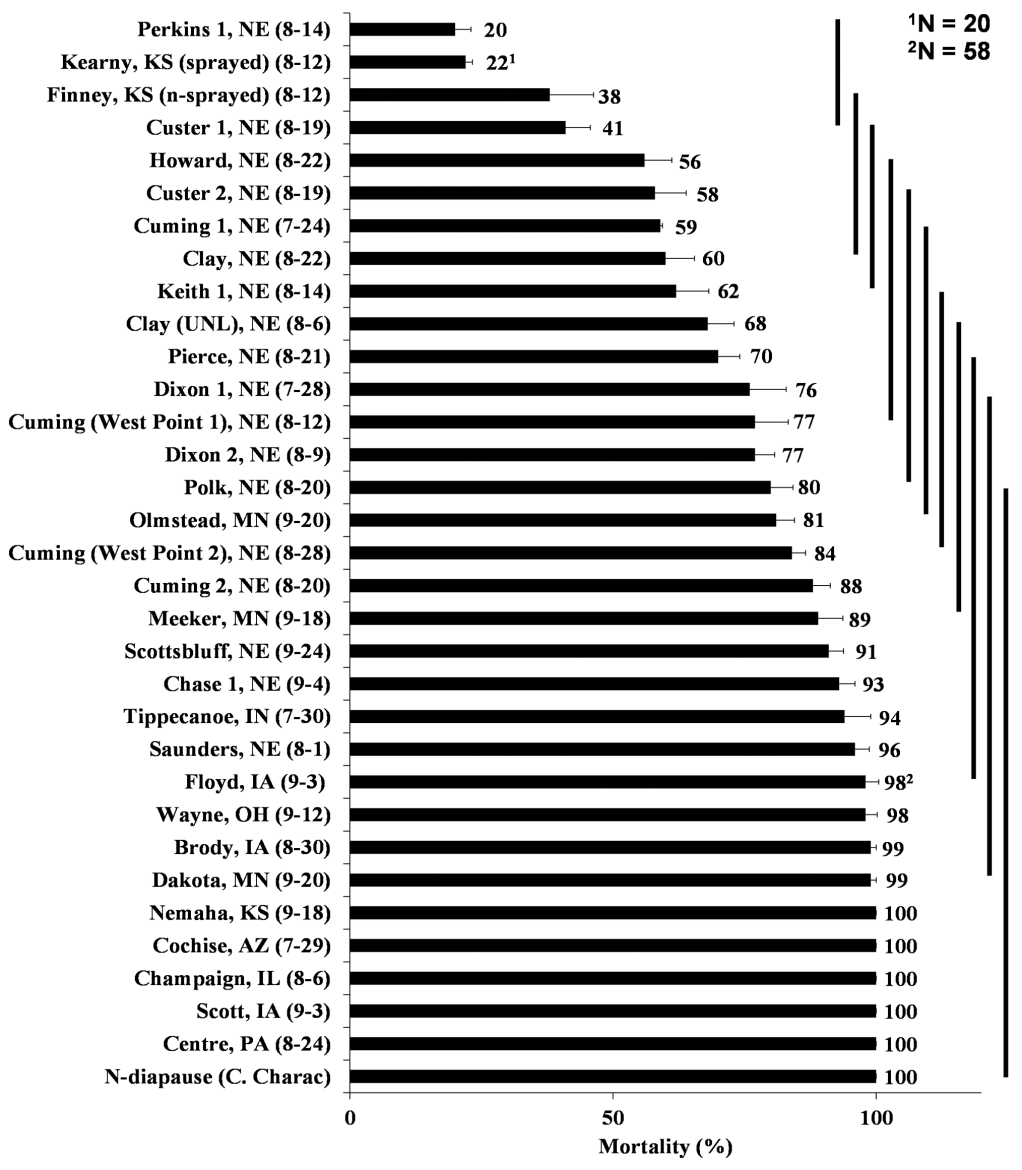
WCR adult mortalities of 32 different field populations from counties throughout the U.S. Corn Belt collected in 2013 (collection date) plus a susceptible laboratory population (non-diapause, Crop Characteristics^®^) after exposure to diagnostic concentration of bifenthrin (0.77 μg of bifenthrin/vial) corresponding to the LC_99_ calculated from 10 WCR lab populations. Means and standard errors are result of 10 replicates (vial), with 10 beetles per vial (unless otherwise stated). Means and standard errors are the result of 10 replicates (vials) with 10 beetles per vial (unless otherwise stated). Population means encompassed by the same solid vertical bars are not significantly different and were compared by least squared means with Tukey adjustment at p ≤ 0.05 using PROC GLIMMIX in SAS 9.3.

**Fig 3:**
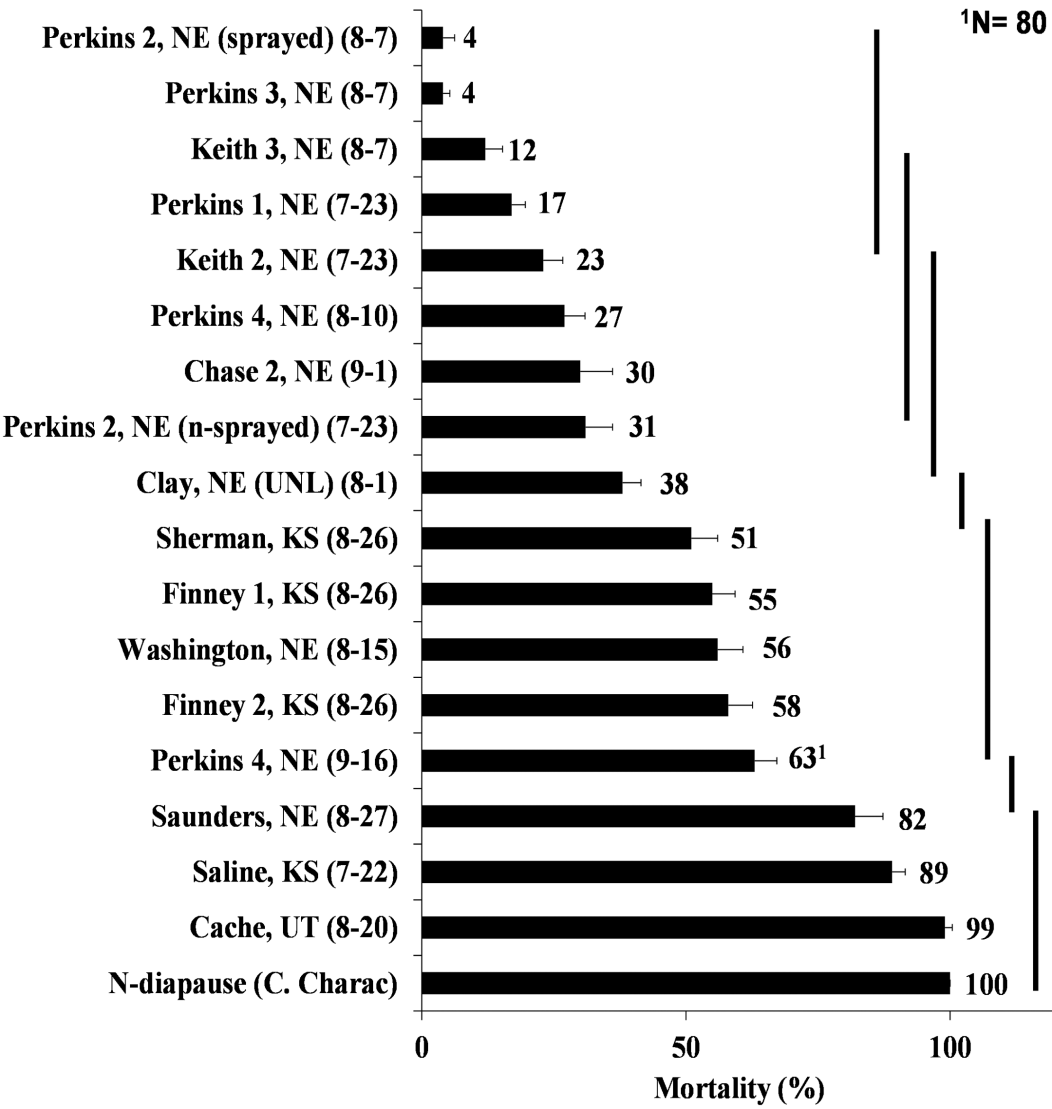
WCR adult mortalities of field populations mostly from several counties in Nebraska and Kansas collected in 2014 (collection date), after exposure to diagnostic concentration of bifenthrin (0.77 μg of bifenthrin/vial). Means and standard errors are the result of 10 replicates (vials), with 10 beetles per vial (unless otherwise stated). Population means encompassed by the same solid vertical bars are not significantly different and were compared by least squared means with Tukey adjustment at p≤ 0.05 using PROC GLIMMIX in SAS 9.3.

Baseline susceptibility was determined using WCR adults from eight different lab populations that were established from field collected adults from throughout the U.S. Corn Belt and maintained in culture for at least 13 years at the USDA/ARS North Central Agricultural Research Lab in Brookings, SD. The baseline assessment also included a non-diapause population (USDA) [42] reared continuously for more than 30 years in the absence of insecticide exposure. A similar non-diapause population from Crop Characteristics LLC^®^ (CCh) (Farmington, MN) was also tested. The pooled analysis of these 10 lab populations was used to estimate a diagnostic concentration based on the LC_99_ that was subsequently used to test field populations (Table 1). Thirty-two adult field populations from 26 different locations in nine states were collected during the summer of 2013 (Figs 1 and 2), and 17 populations from 10 different counties in Nebraska and Kansas, including one population from Utah, were collected during the summer of 2014 (Figs 1 and 3), by using collection devices such as aspirators and sweep nets. The number of adults collected in the fields varied from 200–1000, except for Kearny, KS, and Floyd, IA, where only 20 and 58 beetles were collected, respectively. All adults were delivered or shipped overnight to the University of Nebraska-Lincoln, Insect Toxicology Laboratory and maintained in BugDorm^®^ cages (30 x 30 x 30 cm) (MegaView Science Co., Ltd., Taichung, Taiwan) with artificial diet or fresh sweet corn for 24 h before initiating bioassays. With the exception of one collection in 2013 (Kearny-KS) and another in 2014 (Perkins 1-NE), all adult field populations were collected prior to any adulticide spray. All field collections were allowed access by the owners (private or University) and field collections outside of Nebraska were shipped with APHIS-USDA permissions (No. P526P-13-00045 and P526P-14-03957). The field studies in our research did not involve any endangered or protected species.

**Table 1:** Baseline susceptibility and diagnostic concentration (LC_99_) (± 95% Confidence Interval) of western corn rootworm adults by contact to bifenthrin, combined for overall analysis, from laboratory colonies maintained over several generations.

Populations	N^a^	Slope (±SE)	LC_50_ (95% CI)μg/vial	LC_99_ (95% CI)μg/vial	*X* ^2^ (d.f.)
8 lab colonies +2 n-diapause	2100	2.68 (0.12)	0.10 (0.09–0.13)	0.77 (0.53–1.34)	12.41 (4)

^a^Total number of adults tested

For baseline larval bioassays, eggs from eight of the same lab populations and non-diapause colonies previously described were received and held at 22°C until hatching. The eggs were washed from soil and disinfected with formalin (Formaldehyde 37% w/w, Fischer Scientific, Fair Lawn, NJ) for 3 minutes, rinsed with double distilled water three times, and immersed in a 0.25% solution of methyl 1-(buthylcarbamoyl)-2-benzimidazolecarbamate (Benomyl 98%) (Sigma-Aldrich, St Louis, MO) to minimize fungal growth. The eggs were then transferred to petri dishes with moistened filter paper discs (Whatman^®^ no. 1001 090, Sigma-Aldrich, St Louis, MO) until larval eclosion.

For larval bioassays of field populations, adults of six of the 2013 collections, including five from Nebraska (Perkins, Cuming, Clay, Custer, and Pierce Counties) and one from Kansas (Finney County) (Fig 2) that exhibited increased adult survival in diagnostic bioassays were maintained in BugDorm^®^ cages, provided fresh sweet corn as a food source, and moistened soil (~ 30% volume by weight) as oviposition media. Eggs collected from these field populations were held in 9 cm diameter x 1.4 cm height petri-dishes (Fisher Scientific, Pittsburgh, PA) at 7°C for at least four months in moistened soil (30% v/w) sifted using a #60 mesh sieve (Hogentogler & Co. Inc., Columbia, MD) and then placed in a growth chamber at 22°C to facilitate larval eclosion.

### Baseline susceptibility of WCR adults and larvae

The susceptibility of WCR adults to bifenthrin was determined by exposing beetles to 6–8 increasing bifenthrin concentrations (0.0; 0.0625; 0.125; 0.25; 0.5; 1.0; 2.0; 4.0; 8.0 μg/vial) diluted in acetone and applied to the inside of a 20 ml glass scintillation vial (Thermo Fisher Scientific Inc., Waltham, MA). Technical grade 98% bifenthrin (Chem Service, Inc., West Chester, PA) was diluted in acetone to make a stock solution of 1 μg of bifenthrin/μl of acetone. Each concentration was diluted in acetone and 500 μl of the bifenthrin solution was added to each vial and allowed to dry by rolling on a commercial hotdog roller machine (J.J. Connoly Roll-a-Grill Corp. of America, North Pelham, NY) with the heating element off under a fume hood for 20–30 minutes. For controls, 500 μl of only acetone was added to each vial.

Ten unsexed WCR adults in each population were transferred to each vial with three replicates per concentration. Mortality was recorded after 24 h and compared to control mortality (acetone-only treated vials). Adults that did not respond within 20 seconds to prodding or were unable to right themselves when placed ventral side up were considered dead.

Within colonies, the susceptibility of WCR neonates to bifenthrin was estimated by exposing larvae < 36 h after hatching to 7–8 increasing concentrations of bifenthrin plus control (0.0; 0.03125; 0.0625; 0.125; 0.25; 0.5; 1.0; 2.0; 4.0 ng/cm^2^) applied to filter paper discs (Whatman no. 1001 042, Sigma-Aldrich, St Louis, MO) as described in [43]. The filter papers were placed inside petri dishes (4.7 cm in diameter x 0.7 cm height, Pall Corporation, Port Washington, NY), treated with 150 μl of the insecticide solution diluted in double distilled water (or only double distilled water for control). The insecticide concentrations were prepared from a stock solution (1 μg/μl) as previously described. Treated filter papers were allowed to dry for 5–10 minutes before transferring larvae. Ten to 20 neonates, depending on the number available, were transferred to each petri dish using a fine camel hair paintbrush. Three petri dishes were used per treatment for a total of 30–60 neonates/treatment. The petri dishes were maintained at 23°C and 24 h scotophase. Mortality was recorded 24 h after transfer of neonates to the petri dishes. Neonates that did not move for at least the length of the body after prodding were considered dead. Neonates from field populations used for bioassay in 2014 originated from eggs laid by the same field collected adults that exhibited reduced mortality to the diagnostic concentration in 2013 (Fig 2). The eggs from Kansas populations (Finney, Sherman, and Saline Counties) were collected in 2014 and bioassayed in 2015.

### Diagnostic bioassays for WCR adult field populations

An overall LC_99_ for bifenthrin was estimated from the pooled baseline studies described previously and used as a diagnostic concentration. This concentration of bifenthrin was diluted in 500 μl of acetone from a previously prepared stock solution (1μg/μl), applied into 20 ml scintillated glass vials and dried for 20–30 minutes as previously described. For most populations, at least 110 beetles were included in the diagnostic bioassay with 10 beetles/vial (10 replicates plus control) unless stated otherwise. Ten unsexed WCR adults were transferred to each vial and mortality was recorded after 24 h. Mortality was assessed in the same manner as described previously.

**Fig 4:**
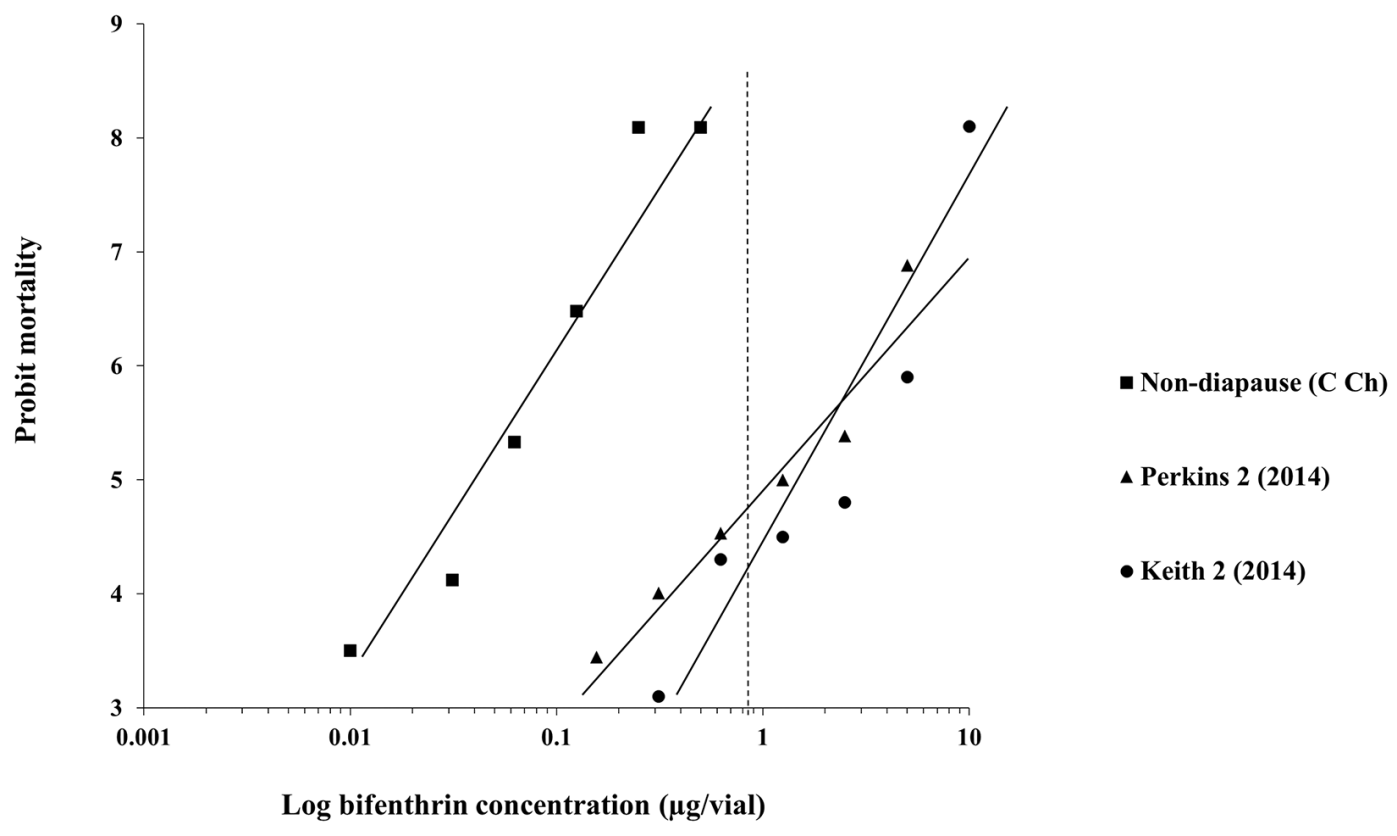
Probit mortality plots of bifenthrin determined by lethal concentration bioassays for adult WCR field populations from Keith and Perkins Counties, NE and a non-diapause laboratory colony (CCh) considered susceptible. Field collections and bioassays performed in 2014. Vertical dash-line represents the diagnostic concentration (LC_99_) of 0.77 μg/vial calculated from the 10 pooled lab colonies (Table 1).

Complete dose-response bioassays for two field populations that exhibited reduced mortality at the diagnostic concentration (Keith 2 and Perkins 2) were conducted to estimate the LC_50_ as previously described and compared with a non-diapause strain (CCh) to determine a resistance ratio (Fig 4).

### Statistical analysis

Only data from bioassays in which control mortality was < 20% were analyzed. The LC_50_ and LC_90_ values were obtained by probit analysis [44] using PoloPlus-PC software [45]. Resistance ratios were calculated by dividing the LC_50_ of the field population by the LC_50_ of the non-diapause susceptible population by using PoloPlus-PC software. Confidence intervals for resistance ratios were calculated by the method in Robertson et al. [46] and compared to test the significance of resistance ratios at the 95% level of confidence (Tables 2 and 3). With this test, if the 95% confidence interval calculated for a ratio does not include 1.0, a significant difference exists between the values being compared [46,47].

**Table 2:** Susceptibility (± 95% Confidence Interval) of western corn rootworm adults by contact to bifenthrin from field populations throughout Nebraska and Kansas, and from two non-diapause laboratory colonies. Resistance ratios compared with lowest non-diapause LC_50_; bioassays conducted in 2014.

Populations	N^a^	Slope (±SE)	LC_50_ (95% CI)μg/vial	RR_50_(95% CI)	LC_90_ (95% CI)μg/vial	RR_90_(95% CI)	*X* ^2^ (d.f.)
**Non-diapause** ^b^	210	4.77 (0.76)	0.54 (0.46–0.64)	-	1.01 (0.82–1.42)	-	1.07 (5)
**Non-diapause** ^c^	240	2.55 (0.28)	0.24 (0.16–0.37)	-	0.77 (0.48–1.85)	-	12.17 (6)
**Clay Co (UNL)-NE** *	240	2.49 (0.28)	0.98 (0.49–2.24)	4.05 (2.97–5.53)	3.20 (1.59–30.45)	4.17 (2.53–6.89)	22.03 (5)
**Perkins Co 2-NE** *	240	1.95 (0.23)	1.14 (0.89–1.49)	4.72 (3.37–6.61)	5.18 (3.49–9.32)	6.75 (3.76–12.2)	4.37 (5)
**Keith Co 2-NE** *	240	1.94 (0.27)	2.20 (1.32–5.12)	9.13 (6.33–13.2)	10.05 (4.54–98.99)	13.10 (6.53–26.3)	10.79 (5)
**Chase Co 2-NE** *	110	2.24 (0.28)	1.38 (0.80–2.96)	5.75 (4.10–7.95)	5.14 (2.54–39.65)	6.70 (3.76–11.9)	14.08 (5)
**Finney Co 1-KS** *	210	2.41 (0.42)	1.43 (0.85–2.32)	5.94 (4.17–8.46)	4.87 (2.84–20.75)	6.35 (3.49–11.6)	7.05 (5)
**Sherman Co-KS** *	210	2.37 (0.28)	0.97 (0.54–1.95)	4.0 (2.92–5.49)	3.35 (1.73–20.70)	4.36 (2.58–7.37)	16.13 (5)

^a^Total number of adults tested

^b^WCR lab colony from USDA/ARS North Central Agricultural Research Laboratory, Brookings, SD

^c^WCR lab colony from Crop Characteristics, Inc., Farmington, MN

*WCR field populations

**Table 3:** Susceptibility (± 95% Confidence Interval) of western corn rootworm neonates by contact to bifenthrin of laboratory colonies from Brookings, SD, including one from a commercial vendor, and from field populations. Resistance ratios compared with lowest non-diapause LC_50_; bioassays conducted in 2014, except Kansas populations were conducted in 2015.

Populations	N^a^	Slope (±SE)	LC_50_ (95% CI)ng/cm^2^	RR_50_(95% CI)	LC_90_ (95% CI)ng/cm^2^	RR_90_(95% CI)	*X* ^2^ (d.f.)
**Wenz**	1456	1.91 (0.11)	0.42 (0.37–0.48)	1.60 (1.36–1.88)	1.97 (1.67–2.38)	2.63 (2.08–3.34)	4.76 (5)
**Kansas**	1452	1.66 (0.10)	0.47 (0.36–0.59)	1.77 (1.48–2.11)	2.75 (2.02–4.19)	3.68 (2.83–4.80)	8.34 (5)
**Linwood**	1482	1.74 (0.09)	0.54 (0.48–0.61)	2.06 (1.75–2.41)	2.97 (2.48–3.67)	3.97 (3.08–5.11)	2.70 (5)
**Dey**	1462	2.24 (0.15)	0.57 (0.46–0.68)	2.16 (1.84–2.55)	2.13 (1.72–2.85)	2.86 (2.27–3.59)	6.50 (5)
**S Dakota**	1452	1.64 (0.10)	0.64 (0.50–0.81)	2.44 (2.05–2.90)	3.87 (2.78–6.13)	5.18 (3.92–6.85)	8.29 (5)
**Penn #1**	1462	1.51 (0.08)	0.65 (0.43–0.97)	2.45 (2.07–2.90)	4.58 (2.57–12.48)	6.13 (4.56–8.23)	25.21 (5)
**Non-diapause** ^b^	960	2.83 (0.16)	0.26 (0.20–0.36)	-	0.75 (0.52–1.33)	-	22.85 (5)
**Non-diapause** ^c^	956	1.74 (0.11)	0.39 (0.32–0.47)	-	2.13 (1.62–3.06)	-	7.49 (6)
**Saline Co-KS** *	400	2.73 (0.37)	0.75 (0.34–1.37)	2.86 (2.27–3.60)	2.22 (1.26–17.81)	2.97 (2.07–4.27)	12.85 (4)
**Sherman Co-KS** *	540	2.85 (0.32)	1.76 (0.87–2.79)	6.66 (5.46–8.14)	4.95 (3.06–20.48)	6.63 (4.94–8.89)	28.40 (6)
**Finney Co 1-KS** *	480	2.39 (0.34)	1.59 (0.96–2.47)	6.03 (4.82–7.55)	5.45 (3.23–25.44)	7.29 (4.83–11.0)	11.75 (5)
**Finney Co 2- KS** *	925	2.24 (0.16)	2.19 (1.58–2.99)	8.31 (7.02–9.85)	8.19 (5.46–16.39)	10.96 (8.34–14.4)	15.30 (5)
**Clay Co-NE** *	1630	1.88 (0.08)	1.16 (0.97–1.38)	4.39 (3.79–5.08)	5.55 (4.25–7.77)	7.42 (5.85–9.42)	10.90 (6)
**Perkins Co 1-NE** *	1410	1.94 (0.11)	1.24 (0.85–1.72)	4.71 (4.00–5.56)	5.96 (3.84–10.38)	7.62 (6.00–9.66)	27.23 (6)
**Perkins Co 2-NE** *	1438	1.87 (0.10)	1.57 (1.24–2.00)	5.97 (5.13–6.95)	7.95 (5.59–13.10)	10.17 (7.89–13.1)	13.08 (5)
**Perkins Co 3-NE** *	960	1.97 (0.13)	0.92 (0.74–1.12)	3.49 (2.90–4.21)	4.12 (3.19–5.75)	5.52 (4.21–7.22)	6.89 (6)
**Custer Co-NE** *	765	1.72 (0.14)	0.76 (0.46–1.10)	2.87 (2.28–3.61)	4.19 (2.72–8.27)	5.61 (4.14–7.60)	15.70 (6)
**Cuming Co-NE** *	480	2.09 (0.21)	0.82 (0.59–1.07)	3.12 (2.47–3.93)	3.38 (2.42–5.59)	4.52 (3.26–6.28)	7.46 (6)
**Pierce Co-NE** *	1864	1.62 (0.07)	0.66 (0.53–0.84)	2.52 (2.17–2.93)	4.11 (2.93–6.48)	5.50 (4.31–7.03)	16.96 (6)
**Chase Co-NE** *	720	1.89 (0.14)	0.78 (0.60–1.01)	2.97 (2.45–3.61)	3.75 (2.59–6.44)	5.01 (3.62–6.93)	7.10 (5)

^a^Total number of neonates tested

^b^WCR lab colony from USDA/ARS North Central Agricultural Research Laboratory, Brookings, SD

^c^WCR lab colony from Crop Characteristics, Inc., Farmington, MN

*WCR field populations

Differences among mortalities of adult WCR obtained from diagnostic bioassays were analyzed by one-way ANOVA in mixed model, using PROC GLIMMIX in SAS software 9.3 [48] where the fixed effects were the different populations. Population mortalities were compared using least squared means with Tukey adjustment at p≤0.05.

## Results

### WCR adult baseline susceptibility

The results of probit analysis of the pooled mortality data from the 10 WCR adult lab populations are presented in Table 1. The LC_50_ of these lab populations varied between 0.05 and 0.20 μg/vial. The pooled data were used to estimate the LC_99_ which was designated as the diagnostic concentration (0.77 μg/vial) (Table 1) and used to screen WCR adult rootworms from field collections in both 2013 and 2014.

### WCR adult susceptibility to diagnostic concentration

There was a large difference in survival at the diagnostic concentration among the 32 field populations assayed in 2013 (Fig 2). Adult mortality ranged from as low as 20% in western Nebraska (Perkins County 1, NE) to 100% in collections from throughout the eastern part of the U.S. Corn Belt as well as the susceptible non-diapause strain (CCh) (Fig 2). Mortality of two field populations collected from western Kansas (Kearny and Finney Counties) was also relatively low (22 and 38%, respectively) in 2013 (Fig 2) although the Kearny County population sample size was small, as mentioned previously. In addition, the beetles from Kearny County were collected after a bifenthrin application for adult control, whereas the Finney County field was not sprayed prior to collection.

The results of 2013 diagnostic bioassays confirmed that reduced bifenthrin susceptibility exists in western Corn Belt collections, especially in southwestern Nebraska and southwestern Kansas, with greater susceptibility in all populations collected east of the Missouri River (Figs 1 and 2). Most populations from western Nebraska exhibited increased tolerance to bifenthrin in 2013, with the exception of Scottsbluff County (91%) and Chase County 1 (93%) (Fig 2). Other Nebraska populations also exhibited reduced susceptibility, such as Cuming County 1 (59%). All populations that exhibited mortality <77% were statistically different from the control which exhibited 100% mortality at diagnostic concentration (Fig 2).

In 2014, we focused most of the field collections in Nebraska and Kansas, especially in those areas where reduced susceptibility was observed in 2013. Similar to 2013 results, reduced mortality was observed at the diagnostic bifenthrin concentration ranging from 4 to 63% in Nebraska and from 51 to 69% in Kansas when compared with the non-diapause WCR colony (CCh) that consistently exhibited 100% mortality in 2014 (Fig 3). One population from eastern Nebraska (Saunders County, Fig 1) exhibited high susceptibility to bifenthrin in 2014 (82%) (Fig 3) which was similar to that observed in 2013 (96%) (Fig 2). Another field population in northeastern Nebraska (Washington County) exhibited intermediate susceptibility at the diagnostic concentration (56%) (Fig 4). A similar trend was observed in Kansas where western populations (Sherman, Finney 1 and Finney 2 Counties) exhibited lower susceptibility (51, 55, and 58%, respectively) as compared to a population from east-central Kansas (Saline County, 89%) (Fig 3). Adult mortalities of field populations in Perkins and Keith Counties were significantly different from those populations that exhibited mortalities above 38% (Fig 3). In addition to the susceptible non-diapause population (CCh), a field population collected in Utah (Cache County) exhibited 99% mortality at diagnostic concentration (Fig 3), again confirming that the reduced susceptibility to bifenthrin is localized in southwestern Nebraska and southwestern Kansas.

In counties where we could obtain collections in both 2013 and 2014, we did not observe a consistent trend from year to year. Some populations exhibited lower mortality in 2014 as compared to 2013 such as Clay-UNL and Saunders Counties, Nebraska (Figs 2 and 3), while Finney County 1, Kansas exhibited higher mortality in 2014 as compared to 2013 (Figs 2 and 3). Field populations from Chase and Keith counties in Nebraska also exhibited variable susceptibility to bifenthrin from 2013 to 2014 (Figs 2 and 3), although the fields collected in 2014 within each county was about 5–10 km distant from the 2013 collections, and may reflect differences in local selective pressures.

In 2014, the adult LC_50_ was estimated for a few of the field populations which ranged from 0.97 (Sherman, KS) to 2.20 μg/vial (Keith 2, NE) (Table 2). All these field populations exhibited higher LC_50_ values when compared to the most susceptible non-diapause strain (CCh) (LC_50_ of 0.24 μg/vial). These field populations were significantly different from the non-diapause strain (CCh), although the resistance ratio was generally <10-fold (Table 2).

### WCR neonate baseline susceptibility

The LC_50_s of WCR neonates to bifenthrin as determined from lab populations ranged between 0.26 ng/cm^2^ (USDA non-diapause) and 0.65 ng/cm^2^ (Pennsylvania #1) (Table 3). The LC_50_s of neonates determined from field populations were all significantly different from the susceptible non-diapause colony (USDA) based on the 95% confidence interval of the resistance ratios (Table 3). The resistance ratios of LC_50_ calculated by comparison of the most tolerant field populations (Finney County 1-KS, 1.59 ng/cm^2^, Finney County 2-KS, 2.19 ng/cm^2^, and Perkins County 2-NE, 1.57 ng/cm^2^) with the USDA non-diapause strain (0.26 ng/cm^2^) ranged between 6 and 9-fold (Table 3) and followed the same trends as the resistance ratios observed for adults from the same populations (Table 2). Saline County-KS exhibited a lower LC_50_ value (0.75 ng/cm^2^) and higher adult mortality (89%) at diagnostic concentration (Table 3; Fig 3) when compared to the other populations in Kansas.

### Validating diagnostic concentration

The concentration response curves for adult WCR field populations from Keith County 2 and Perkins County 2 were plotted to show the difference in susceptibility of each population when compared to the non-diapause laboratory strain (CCh) considered susceptible to bifenthrin (Fig 4). The resistance ratios of Keith 2 and Perkins 2 populations were around 10 and 6-fold, respectively, when compared to the non-diapause strain (CCh) (Table 2; Fig 4). In both populations, the diagnostic concentration was estimated to produce mortality approximating 20–30% (and 100% in the susceptible non-diapause population, Fig 3). These results are consistent with the estimated LC_50_s of the same field populations for both neonates and adults (Tables 2 and 3; Figs 2 and 3).

## Discussion

The results of this investigation suggest that resistance to bifenthrin is evolving in populations of western corn rootworms in southwestern areas of both Nebraska and Kansas. The results of adult diagnostic bioassays indicate that all populations outside of Nebraska and Kansas exhibited susceptibility similar to that of the susceptible non-diapause laboratory population (CCh) indicating that reduced susceptibility is limited in distribution and has not moved beyond western Nebraska and southwestern Kansas. The high susceptibility of populations east of the Missouri River is consistent with the generally reduced use of chemical insecticides for both larval and adult control and greater reliance on crop rotation to manage rootworm populations in the eastern Corn Belt. In the western Corn Belt where irrigation, confined livestock operations and ethanol production make continuous corn production the most economic production practice for many growers, a reliance on chemical control practices has developed. This is evidenced by the initial occurrence in Nebraska of both cyclodiene resistance and methyl-parathion resistance in areas where continuous corn production was a common practice [20,21,30].

Although the adult LC_50_s estimated for Perkins 2 and Keith 2 populations indicate that the level of resistance is relatively low (<10-fold), a similar level of resistance was observed in larval progeny obtained from Perkins County 2 field-collected adults. Reduced susceptibility of both adults and their larval progeny support the presence of a heritable trait [49]. The larval LC_50_ values observed for the Finney 1 and Sherman populations (1.59 and 1.76 ng/cm^2^, respectively) were significantly greater than the LC_50_ for neonates of the susceptible non-diapause strain CCh (0.26 ng/cm^2^) (Table 3), and the resistance ratios (RR = 6.03 and 6.66, respectively) were similar to the resistance ratios observed in adult bioassays of these same populations (RR = 5.94 and 4.0, respectively). These results support the conclusion that reduced efficacy observed in southwestern Nebraska and southwestern Kansas are related to resistance evolution.

The WCR adult LC_50_ of bifenthrin (0.10 μg/vial) estimated from the pooled laboratory populations (Table 1) is similar to the LC_50_s determined for WCR adults to bifenthrin from field populations performed by Zhu et al. [33] between 1997 and 2002 in several counties in southwestern Kansas including Finney County. In this study, adult LC_50_ values for bifenthrin ranged from 0.089 to 0.13 μg/vial using similar methodology, except that mortality was evaluated after 6 h. Zhu et al. [33] also reported bifenthrin to be the most lethal insecticide tested when compared with other rootworm insecticides such as methyl parathion, carbaryl, fipronil, and another pyrethroid, cypermethrin. Therefore, the reduction in susceptibility to bifenthrin in our study suggests that the changes in bifenthrin susceptibility likely arose since 2005.

Meinke et al. [30] also reported WCR adults from Nebraska to be susceptible to bifenthrin, with similarly low LD_50_s among different field populations when compared to methyl parathion and carbaryl, which exhibited resistance ratios as high as 16-fold and 9-fold, respectively. These results were obtained by topical bioassays of adults rather than the residual bioassays used in the present study. Although bifenthrin resistance was not detected, the highest LD_50_ values reported by Meinke et al. [30] were in the same geographical areas where methyl parathion and carbaryl resistance were identified, suggesting the potential for cross-resistance among insecticide classes. It is possible that the reduced susceptibility to bifenthrin observed in our study could have resulted from cross-resistance to compounds previously used in adult management programs, as organophosphate and carbamate resistance has been previously demonstrated to be highly persistent [26]. However, in areas of southwestern Nebraska and southwestern Kansas where reduced bifenthrin susceptibility was detected, there are no previous reports of resistance or control failures similar to those observed in other areas of Nebraska [50,51].

In eastern Nebraska, susceptibility to bifenthrin was variable, which may relate to the cross-resistance previously described [30,52] or variability in local selection pressure leading to different frequencies of resistant alleles across the landscape. Local variability within the same counties was observed in 2013 (Cuming and Custer Counties, NE), in which some of the collection sites (Fig 1) were separated by relatively short distances (30 km). Variability on a larger spatial scale was also observed in Kansas, as 100% mortality at the diagnostic concentration was observed in the eastern-most collection (Nemaha County) in 2013 in contrast to <40% mortality in southwestern Kansas (Figs 1 and 2). In addition, variation within the same growing season was observed in 2014. In one of the fields in Perkins County (Perkins 4), beetles were collected early and late in the season and mortality at the diagnostic concentration increased from 27% to 63% (Fig 3). This increased susceptibility might be associated with the movement of beetles from areas where WCR is still susceptible. Adult distributions within and among fields can change over time especially if plant phenology contrasts occur as adult food becomes more limiting later in the season [53,54]. In a second field, also from Perkins County (Perkins 2), collections were performed before and after application of bifenthrin for adult control and mortality at the diagnostic concentration decreased from 31% to 4% (Fig 3) suggesting an increase in frequency of resistant individuals associated with the application of insecticide. Various factors can affect variation in bioassay results so it is difficult to determine whether local differences or year-to-year variation represents significant changes in susceptibility.

In spite of this variation the results from 2014 support the 2013 results and collectively they present a consistent general picture in which WCR populations from the western part of both Nebraska and Kansas are more tolerant to bifenthrin than eastern populations indicating that resistance is still localized and in early stages of development. However, it should be noted that localized selection pressure, distance to susceptible populations, migration, genetic variability, and time of sampling during the season all can affect susceptibility. Additional sampling and bioassays will be necessary to more accurately assess the distribution of bifenthrin resistance at different scales in local areas within and among counties.

It is unclear what is driving bifenthrin resistance evolution in southwestern areas of Nebraska and Kansas, but it is likely a combination of factors. Areas of western Nebraska and Kansas have a long history of aerial application of pyrethroid insecticides for adult control [25]. In addition, bifenthrin is commonly used as a foliar treatment for western bean cutworm, *Striacosta albicosta* (Smith) and as a miticide to control two spotted spider mites, *Tetranychus urticae* Koch, and banks grass mites, *Oligonychus pratensis* (Banks). Although WCR is not the target pest in this case, selective pressures against rootworm adults are likely since these applications are routinely used during periods of WCR adult activity. In addition, for some of the fields identified from Perkins County, bifenthrin has been applied both at-planting as a soil insecticide as well as foliar application for adult control for at least five consecutive years. Moreover, because bifenthrin is used as a soil insecticide, adults collected from some fields may have included survivors that completed development after larval exposure. Given that pyrethroid insecticides are applied for both larval and adult management, it is not clear whether the change in susceptibility resulted from selection against larvae, adults or potentially both stages of development. However, because all previous documented cases of WCR resistance to insecticides have occurred when population management was practiced (i.e., selection of the majority of the population with repeated use of broadcast applications) and WCR resistance has not been documented to any soil insecticide applied in furrow or in narrow bands over the row (includes built-in refuge), this suggests that the long tradition of aerial application in western areas of Nebraska and Kansas may have played an important role.

WCR resistance to insecticides has long been reported since the late 1950s, and pyrethroid resistance represents the fourth major class of chemical insecticides, along with cyclodienes, organophosphates, and carbamates in the past 60 years for corn rootworm control for which resistance appears to be affecting efficacy. In addition, rootworms have documented resistance to crop rotation and to Bt maize, and its capacity for resistance evolution approaches another chrysomelid beetle, the Colorado potato beetle (CPB), *Leptinotarsa decemlineata* (Say), in terms of resistance potential, which has also evolved resistance to the four major insecticide classes, in addition to developing a behavioral resistance [55]. As a consequence, WCR will continue to pose serious pest management challenges. Pyrethroids represent one of the few remaining classes of chemical insecticides for rootworm control. As soil insecticides and adulticides are being recommended in best management practices in areas where resistance to transgenic maize has been documented, it is imperative that sustainable pest management approaches that incorporate multiple management tactics be implemented for western corn rootworm control. Averting widespread resistance to pyrethroids is therefore critically important to the development of sustainable rootworm management approaches.

In summary, the results of this research suggest that resistance of WCR field populations to bifenthrin is emerging and evolving in areas west of the Missouri River, especially in southwestern regions of both Nebraska and Kansas, which historically have used bifenthrin for larval control during planting, as well as for adult control to reduce oviposition of females. Future field efficacy studies with formulated pyrethroid products in areas where resistance occurs will help clarify how evolving resistance levels may contribute to rootworm injury in cornfields. Additional studies of cross-resistance and inheritance as well as continued surveillance of field populations in those problem areas within each state will provide a better understanding of the nature of this resistance and its potential to spread to other corn growing regions.
